# Unraveling the impact of pH, sodium concentration, and medium osmolality on *Vibrio natriegens* in batch processes

**DOI:** 10.1186/s12896-024-00897-8

**Published:** 2024-09-23

**Authors:** Eva Forsten, Steffen Gerdes, René Petri, Jochen Büchs, Jørgen Magnus

**Affiliations:** AVT- Biochemical Engineering, RWTH Aachen University, Forckenbeckstraße 51, 52074 Aachen, Germany

**Keywords:** *Vibrio natriegens*, oxygen transfer rate, maximum growth rate, osmolality, MOPS buffer, small-scale cultivation

## Abstract

**Background:**

*Vibrio natriegens*, a halophilic marine γ-proteobacterium, holds immense biotechnological potential due to its remarkably short generation time of under ten minutes. However, the highest growth rates have been primarily observed on complex media, which often suffer from batch-to-batch variability affecting process stability and performance. Consistent bioprocesses necessitate the use of chemically defined media, which are usually optimized for fermenters with pH and dissolved oxygen tension (DOT) regulation, both of which are not applied during early-stage cultivations in shake flasks or microtiter plates. Existing studies on *V. natriegens’* growth on mineral media report partially conflicting results, and a comprehensive study examining the combined effects of pH buffering, sodium concentration, and medium osmolality is lacking.

**Results:**

This study evaluates the influence of sodium concentration, pH buffering, and medium osmolality on the growth of *V. natriegens* under unregulated small-scale conditions. The maximum growth rate, time of glucose depletion, as well as the onset of stationary phase were observed through online-monitoring the oxygen transfer rate. The results revealed optimal growth conditions at an initial pH of 8.0 with a minimum of 300 mM MOPS buffer for media containing 20 g/L glucose or 180 mM MOPS for media with 10 g/L glucose. Optimal sodium chloride supplementation was found to be between 7.5 and 15 g/L, lower than previously reported ranges. This is advantageous for reducing industrial corrosion issues. Additionally, an osmolality range of 1 to 1.6 Osmol/kg was determined to be optimal for growth. Under these optimized conditions, *V. natriegens* achieved a growth rate of 1.97 ± 0.13 1/h over a period of 1 h at 37 °C, the highest reported rate for this organism on a mineral medium.

**Conclusion:**

This study provides guidelines for cultivating *V. natriegens* in early-stage laboratory settings without pH and DOT regulation. The findings suggest a lower optimal sodium chloride range than previously reported and establish an osmolality window for optimal growth, thereby advancing the understanding of *V. natriegens’* physiology. In addition, this study offers a foundation for future research into the effects of different ions and carbon sources on *V. natriegens*.

**Supplementary Information:**

The online version contains supplementary material available at 10.1186/s12896-024-00897-8.

## Background

*Vibrio natriegens* is a gram-negative, facultatively anaerobic, marine γ-proteobacterium with no known pathogenicity factors [1, 2]. Its generation time of 9.8 min on complex medium is about half that of *Escherichia coli* [1, 3], making it one of the fastest-growing non-pathogenic organisms known [4]. As its very high growth rate promises higher space–time-yields, the organism is being investigated as a new biotechnological workhorse. Many metabolic engineering tools have been developed and published for *V.* *natriegens* [5–9]. Using these tools, the organism is currently explored for a variety of biotechnological applications, including the biomanufacturing of proteins [5, 10–14], biofuels [15, 16], organic acids [17, 18], amino acids [19], terpenoids [20], and other valuable products [21, 22]. Overall, research and applications are expected to increase in the next years [4].

So far, exceptionally high growth rates of *V.* *natriegens* have mainly been described on complex media, where generation times of < 10 min or growth rates of up to 4.4 1/h have been reported for short periods of cultivation time [1, 17]. Due to unavoidable batch-to-batch variability, the composition of complex components can vary, resulting in inconsistent process stability and performance on complex media [23–26]. To ensure consistent processes and product quality, chemically defined media are required. The literature describes various mineral media on which cultivations of *V. natriegens* have been successfully performed [5, 14, 17, 27]. Growth rates achieved during *V.* *natriegens* cultivations on mineral media (37 °C, glucose as carbon source) range from 1.36 1/h on mineral salt medium [28], 1.66 1/h on VN medium [17] and 1.7 1/h on M9 medium [27].

A mineral medium for *V. natriegens* should meet a number of nutritional requirements. As a halophilic organism, *V. natriegens* requires sodium, although the literature is discordant about the optimal concentration: Depending on the study, 15 – 25 g/L NaCl is recommended [5, 14, 28, 29]. Sodium is typically supplied in the form of sodium chloride, but recently, a low-chloride medium composition has been reported as an alternative [30]. While the osmoregulation of *V.* *natriegens* is still poorly understood, hypo-osmotic stress has been reported as a negative influence [31]. Consequently, it is unsurprising that *V.* *natriegens* tolerates high-osmolality conditions well [29]. Another important consideration is the medium pH. Generally, *V.* *natriegens’* growth optimum lies around a neutral pH of 7.5 [1]. During growth on glucose, *V. natriegens* secretes 25% of the available carbon as acetate via its overflow metabolism, even at conditions of sufficient oxygen availability [32]. The acetate can be taken up and metabolized once the glucose is depleted [17, 33]. Before this occurs, the pH of the medium decreases, necessitating either pH buffering or regulation [33]. While Payne et al. [2] investigated the influence of the starting pH in complex medium on *V. natriegens*, there is a distinct lack of research when it comes to mineral media. Existing studies usually restrict their investigation to one pH-buffered and an unbuffered condition, as well as a limited number of sodium concentrations at either the pH-buffered or unbuffered condition [17, 28, 33]. Furthermore, the influence of the medium osmolality, which is affected by both the pH buffer and sodium concentration, has been fully disregarded up until now. To determine a global optimum of pH buffering, sodium concentration, and osmolality, a combined study of the three parameters is needed.

The initial steps of process development, such as strain and enzyme screening, are usually carried out in small scale, where cultivations are often performed unregulated and without the use of online monitoring techniques [34]. As mineral media are often designed for fermenters equipped with pH and DOT control, suboptimal cultivation conditions in microtiter plates and shake flasks frequently go unnoticed. They only become apparent once the process is upscaled to fermenter scale: The most promising strategy for an exact process transfer is to distribute the same batch of inoculated medium into MTPs and fermenters and running the both cultivations without pH and DOT [35, 36]. This may be achieved by sterilizing and inoculating the medium in a fermenter, and subsequently distributing aseptically sampled fermentation broth to a MTP for parallel cultivation. If suboptimal conditions were not addressed during small-scale process development, they may become apparent only after scale-up. A strategy to generate much earlier insight into unregulated small-scale processes is facilitated by novel devices, such as the Respiration Activity Monitoring System (RAMOS) [37, 38] and micro(µ)-scale Transfer rate Online Measurement device (µTOM) [39]. These devices enable the online monitoring of the oxygen transfer rate (OTR) in shake flasks and microtiter plates. This study represents the first application of both technologies to investigate *V. natriegens*. The OTR is a widely used parameter for assessing the physiological condition of aerobic cultures [40–42] since it allows the identification of the growth phase, the quantification of the growth rate [43] as well as the detection of limiting or inhibiting effects [44–46] all from a single signal. This approach reduces sampling while providing a high information content [36] and has been applied to develop and optimize media for a range of microorganisms in the past [26, 45, 47, 48].

In this study, the OTR was used to evaluate the effect of the medium composition on the growth of *V.* *natriegens* on glucose, focusing on sodium concentration, pH buffering, and the medium osmolality. A screening approach was chosen to identify a global optimum of the maximum growth rate depending on the medium composition. Monitoring the OTR in microtiter plates allowed to conduct the screening directly under the unregulated small-scale cultivation conditions for which the medium is intended. The OTR data also allows for early detection of problems with the cultivation and, thus, serves as preparation for a later scale up into lab-scale fermenters. In addition, the time-resolved online data allowed insight into growth indicators, such as the time point of reaching the maximum growth rate, the time of glucose depletion, as well as the onset of the stationary phase. This study aims to provide a reference for other groups working with *V.* *natriegens* since the medium can be tailored for any specific purpose.

## Methods

### Microorganism

*Vibrio natriegens* Vmax pET19b::LevS1417 was kindly provided by Prof. Dr. Deppenmeier’s group at the University of Bonn, Germany. Competent *Vibrio natriegens* Vmax X2 (*V.natriegens* ATCC 14048 dns::LacI-T7-RNAP) cells were originally obtained from Biocat GmbH (Heidelberg, Germany). This organism is from here on referred to as *V. natriegens* Vmax. The heterologous production of the levansucrase LevS1417 (GenBank accession: KXV23964.1) has previously been described by Hövels et al. [49]. In this study, the lac-controlled expression of LevS1417 was not induced.

### Media

If not otherwise noted, all chemicals were acquired from Carl Roth GmbH + Co. KG (Karlsruhe, Germany).

Brain heart infusion (BHI) supplemented with v2-salts was used for precultures, as recommended by Weinstock et al. [5]. BHI (1.25 × concentrated) and v2-salts (5 × concentrated) were separately autoclaved, stored at room temperature, and then combined for each preculture. The combined BHI + v2 contained 37 g/L powdered Bacto™ Brain–Heart Infusion (Article 237500, Becton Dickinson GmbH, Heidelberg, Germany), 11.9 g/L NaCl, 0.3 g/L KCl, and 4.7 g/L MgCl. To ensure plasmid stability, 100 µg/L carbenicillin from a 1000 × concentrated stock solution (sterile filtered and stored at -20 °C) was added immediately before use. In this work, expression of the heterologous protein was not induced.

Wilms-MOPS medium [50] was used in various modified compositions during this study. The following stock solutions were prepared and stored separately. The 4 × concentrated main salt solution contained 27.92 g/L (NH_4_)_2_SO_4_, 12 g/L K_2_HPO_4_, and 8 g/L Na_2_SO_4_ (pH adjusted to 7.5 with NaOH before autoclaving, stored at room temperature); the 25 × concentrated glucose solution contained 550 g/L glucose · H_2_O (equates 500 g/L glucose; autoclaved and stored at room temperature); the 100 × concentrated MgSO_4_ stock solution contained 50 g/L MgSO_4_ · 7 H_2_O (autoclaved, stored at room temperature); the 1000 × concentrated thiamin stock solution contained 10 g/L thiamin-HCl (sterile filtered, stored at 4 °C). The 1000 × concentrated trace element solution contained 0.54 g/L ZnSO_4_ · 7 H_2_O (Merck KGaA, Darmstadt, Germany), 0.48 g/L CuSO_4_ · 5 H_2_O (Merck KGaA, Darmstadt, Germany), 0.3 g/L MnSO_4_ · H_2_O, 41.76 g/L FeCl_3_ · 6 H_2_O, 1.98 g/L CaCl_2_ · 2 H_2_O, 33.4 g/L Na_2_EDTA · 2 H_2_O (Merck KGaA, Darmstadt, Germany), and 0.54 g/L CoCl_2_ · 6 H_2_O (sterile filtered, stored at 4 °C protected from light).

For the medium modifications, a 200 g/L NaCl stock solution (autoclaved, stored at room temperature) and a 2 M 3-(*N*-Morpholino)-propane sulfonic acid (MOPS) stock solution (418.5 g/L MOPS (acid form), pH set to 7.5 or 8.0 with KOH), sterile filtered and stored at room temperature) were prepared.

Per 1 L of modified Wilms-MOPS medium (15 g/L NaCl added to the standard configuration), 250 mL main salt solution, 40 mL glucose solution, 10 mL MgSO_4_ solution, 1 mL thiamin solution, 1 mL trace element solution, and 1 mL carbenicillin solution were combined with 100 mL of MOPS solution and 75 mL NaCl solution and added to 522 mL of deionized water. The water volume was reduced accordingly in experiments with varied MOPS and NaCl concentrations. Less water (e.g., 500 mL) was added for cultivations, and the medium was filled up after inoculation to the final volume of 1 L.

Per 1 L of half-concentrated modified Wilms-MOPS medium, only 125 mL main salt solution, 20 mL glucose solution, 5 mL MgSO4 solution, 0.5 mL thiamin solution, 0.5 mL trace element solution, and 0.5 mL carbenicillin solution were combined with a varied volume of MOPS solution (pH set to 7.5 using NaOH) and NaCl solution and added to 250 mL of deionized water. After inoculation, the medium was filled up with deionized water to its final volume of 1 L. For the experiments with increased initial pH, the pH of the MOPS buffer was set to 8.0 using KOH before sterile filtration; the rest of the medium was prepared identically to the half-concentrated modified Wilms-MOPS medium, as described above.

### Shake flask cultivations in a RAMOS device

In anticipation of elevated oxygen demand due to the rapid substrate uptake and growth rate of *V. natriegens*, a high maximum oxygen transfer capacity (OTR_max_) was prioritized. According to Meier et al. [51], 8 mL filling volume and a shaking frequency of 350 rpm were expected to allow a theoretical OTR_max_ of approx. 90 mmol/L/h for BHI + v2 and approx. 65 mmol/L/h for Wilms-MOPS medium. The difference is due to the difference in medium osmolality.

For all shake flask cultivations, 250 mL RAMOS or Erlenmeyer flasks were filled with 8 mL of inoculated medium and incubated on an orbital shaker (ISF1-X, Kühner AG, Birsfelden, Switzerland) at 37 °C, 350 rpm shaking frequency, and a shaking diameter of 50 mm. RAMOS flasks are modified Erlenmeyer flasks that can be connected to a RAMOS device, which enables the measurement of the oxygen transfer rate (OTR), carbon dioxide transfer rate (CTR), and respiratory quotient (RQ) in shake flasks [37, 38]. In this study, an in-house built RAMOS device was used with the following settings: 10 min flush phase, 4 min measurement phase, 1 min high-flow phase. Thus, one measurement value for OTR, CTR, and RQ was obtained every 15 min.

For each preculture, BHI + v2-medium was inoculated from a cryo culture to an initial optical density (OD_600_) of 0.05. The culture was incubated using the shake flask cultivation settings above. The preculture was harvested once the OTR reached a value of > 80 mmol/L/h, which took approximately 3.5–4 h and corresponded to a final OD_600_ of roughly 10.

For main cultures with offline sampling, Wilms-MOPS medium with 200 mM MOPS buffer and 15 g/L NaCl was inoculated to OD_600_ = 0.25 from a preculture. The inoculated medium was thoroughly mixed and distributed to 250 mL RAMOS flasks and standard 250 mL Erlenmeyer flasks with cellulose plugs (Culture plug ROTILABO®, Carl Roth GmbH + Co. KG, Karlsruhe, Germany). All flasks were incubated in the same incubation hood using the shake flask cultivation settings above. While the RAMOS flasks were connected to an in-house built RAMOS device, the Erlenmeyer flasks were used for offline sampling. At each sampling point, an Erlenmeyer flask was extracted without halting the shaker to ensure minimal sampling interference with the ongoing cultivation. After analyzing the sample, the residual culture broth of the respective flask was discarded, and the flask not placed back onto the shaker.

### Microtiter plate cultivations in a µTOM device

All microtiter plate cultivations were conducted in 96-well microtiter plates (J.T.Baker®, Plate Medio (2 mL), VWR International GmbH, Darmstadt, Germany). The cultivations were prepared in four steps: In the first step, a double concentrated NaCl/MOPS gradient was pipetted into a master mix plate at 500 µL per well. In the second step, all other medium components were combined at double concentration and inoculated to OD_600_ = 0.5 from a preculture. In the third step, the double-concentrated inoculated medium was added at 500 µL per well to the master mix plate. Thus, the NaCl/MOPS gradient and all other medium components were diluted to their respective target concentration, and the biomass was consequently diluted to OD_600_ = 0.25. The fourth and last step consisted of mixing each well by pipetting up and down with a multichannel pipette and transferring 50 µL per well from the master mix plate to a separate cultivation plate. After this step, the remaining volume of the master mix plate was sampled immediately for HPLC and osmolality measurements.

The cultivation plate was placed in a µTOM device [39] (prototype provided by Kuhner Shaker GmbH, Herzogenrath, Germany) on an orbital shaker and incubated at 37 °C, 1000 rpm shaking frequency, and 3 mm shaking diameter. Analogous to the RAMOS technology, the µTOM device relies on a two-phase measurement cycle. During the flush phase, the microtiter plate is located at the bottom of the cultivation chamber and aerated at a specific gassing rate. At the beginning of the measurement phase, the microtiter plate is pressed upwards against the lid of the chamber by a lifting mechanism, which seals off the individual wells. An optoelectronic sensor module in the lid detects the decrease in oxygen partial pressure during the measurement phase. From this signal, the oxygen transfer rate can be calculated. At the end of the measuring phase, the microtiter plate is lowered to the original position by the lifting mechanism, and the next measuring cycle begins with a flush phase. For this study, the device was set to 10 min flush phase and 5 min measurement phase, which resulted in one OTR measurement value every 15 min. To reduce evaporation, air with a relative humidity of > 80% was used for gassing; the plate was weighed before and after each cultivation to monitor the evaporation. Before endpoint sampling, 450 µL deionized water was added per well (tenfold dilution) to ensure sufficient sampling volume.

### Offline analysis

The optical density (OD_600_) was determined at a wavelength of 600 nm via a Genesys 20 photometer (Thermo Scientific, Dreieich, Germany). Before measurement, the culture broth was diluted to values between 0.1 and 0.3 using a 9 g/L NaCl solution. pH measurements were conducted at room temperature using a pH meter (HI 221, Hanna Instruments, Germany).

Osmolality and HPLC samples were pretreated by a centrifugation step (1500 rpm, 10 min, room temperature) in either a ROTINA 35R (only microtiter plates, Andreas Hettich GmbH & Co. KG, Tuttlingen, Germany) or a Sigma 1–15 centrifuge (only reaction tubes, Sigma Laborzentrifugen GmbH, Osterode am Harz, Germany). Samples in microtiter plates were vacuum-filtered through 96-well filter plates (AcroPrep™ Advance 96 Filter Plate 0.2 µm Supor, Pall Life Sciences, Dreieich, Germany), while larger sample volumes were filtered through syringe filters (Rotilabo syringe filter cellulose acetate 0.2 µm, Carl Roth GmbH + Co. KG, Karlsruhe, Germany).

Medium osmolality was measured with a cryoscopic osmometer (Osmomat 3000 Basic, Gonotec GmbH, Berlin, Germany). Glucose and acetate concentrations were determined using a Prominence HPLC (Shimidazu Europe, Düsseldorf, Germany) with an organic acid column (300 × 8 mm, ROA-Organic Acid H +, Phenomenex, Aschaffenburg, Germany) at 75 °C and a carrier flow of 0.8 mL/min 0.25 mM sulfuric acid.

### Maximum growth rate calculation from the OTR

All growth rate calculations were performed in MATLAB® (Mathworks, Inc.). According to Stöckmann et al. [43], the general relationship between the OTR and the growth rate is calculated by Eq. (1).

1 $$\upmu =\frac{\text{ln}\left({OTR}_{t}\right)-\text{ln}({OTR}_{{t}_{n}})}{t-{t}_{n}}$$

The growth rate itself is influenced by several factors, including temperature, carbon source, medium osmolality, and pH [52]. While temperature and carbon source are usually kept constant, osmolality and pH can change during batch cultivations. In particular, a decrease in osmolality due to glucose and nitrogen uptake increases the growth rate, whereas a decline in pH decreases it.

For a consistent and reproducible calculation of the maximum growth rate across all experiments, calculations were performed on a subset of the OTR data (see Fig. S1A). Temperature effects at the beginning of cultivation were prevented from influencing the calculations by excluding the first 30 min of cultivation time, corresponding to the first two OTR measurement points. Similarly, to ensure that the growth rate was calculated for growth on the same initial carbon source, only the OTR until the glucose batch peak was considered. In the next step, the instantaneous growth rate for this reduced data set was determined by applying a sliding lin-log fit according to Eq. (1) over five consecutive OTR measurement values (corresponding to 1.0 h cultivation time, demonstrated in Fig. S1B). Only fits with an R^2^ value greater than 0.9 were further considered (Fig. S1C). The maximum growth rate was then identified as the highest instantaneous growth rate obtained (illustrated in Fig. S1B).

R^2^ cutoffs of 0.99, 0.98, and 0.95 were also considered but did not yield the same biologically probable results. Similarly, the fit was performed for three/four consecutive values and was not found to be as consistent, as small deviations of the measurement had a much greater influence on the fit (compare Fig. S2). Performing the fit for six values at a time was ruled out because a fit duration of > 1.0 h was considered too long to adequately assess the instantaneous growth of a fast-growing microorganism.

### Replicates and biological variability

The overall biological variability was estimated through a series of four experiments with independent precultures (data provided in Fig. S3). Between shake flask and MTP experiments, the relative standard deviation of µ_max_ was determined as low as 6.6%.

The goal of this study was to determine the global optimum of the cultivation conditions. To ensure that not merely local optima were observed, the microtiter plate experiments were conducted with the highest possible throughput. Thus, replicate cultivations were not conducted at the same experimental conditions, but instead evenly spaced out between the conditions. This allowed for more information to be generated while still enabling to detect issues and outliers by drawing a trend across neighboring conditions.

## Results

### State of the art cultivation in shake flasks

**Fig. 1:**
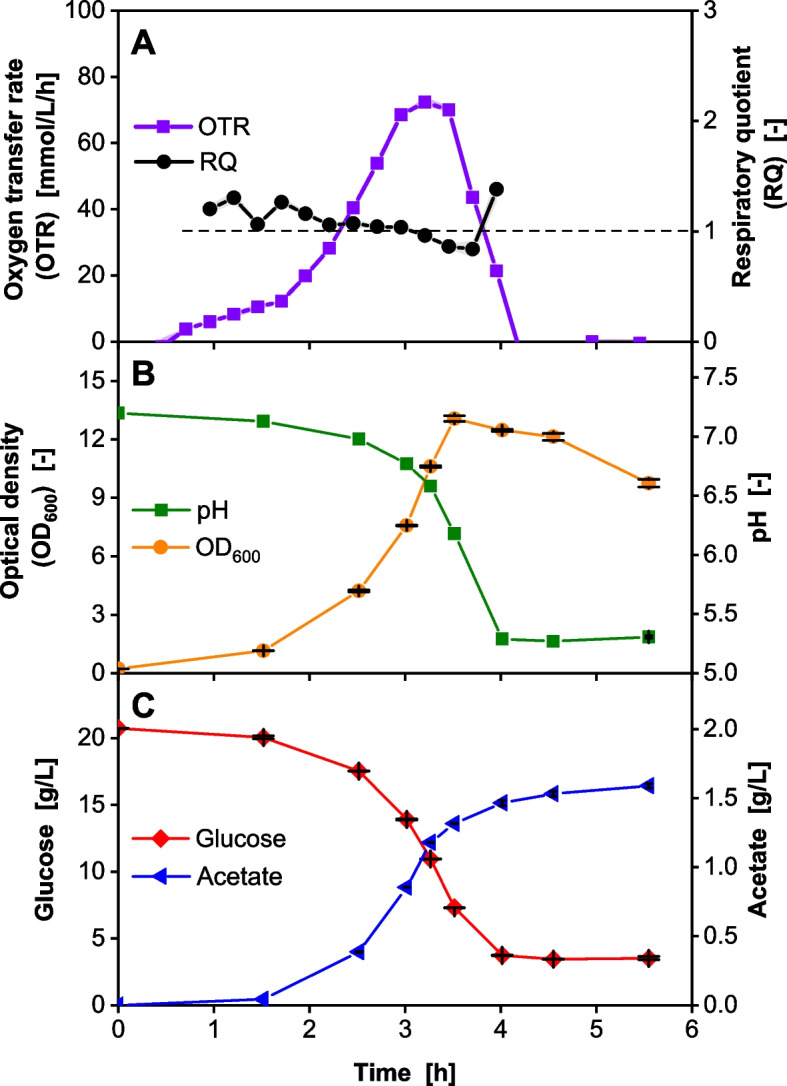
Shake flask cultivation of *Vibrio natriegens* in Wilms-MOPS medium with 15 g/L NaCl *V. natriegens* Vmax pET19b::LevS1417 in modified Wilms-MOPS medium (20 g/L glucose, 15 g/L NaCl, 200 mM MOPS buffer set to pH 7.5). Initial OD_600_ 0.25, 37 °C, 8 mL filling volume in 250 mL flask, 350 rpm at 50 mm shaking diameter. **A** Oxygen transfer rate and respiratory quotient monitored using a RAMOS device. Mean of *n* = 2 replicates shown; shadows indicate minimum/maximum and are barely visible due to the very low deviation. For clarity, the RQ is only shown for OTR values > 5 mmol/L/h. **B** Offline sampling results: OD_600_ and pH. Error bars depict the standard deviation of *n* = 3 replicates. **C** Glucose and acetate concentrations were determined offline via HPLC analysis. Error bars depict the standard deviation of *n* ≥ 3 replicates. In some cases (i.e., acetate), the error bars are smaller than the symbols

While existing literature presents conflicting information regarding the NaCl concentration optimum for *Vibrio natriegens*, 15 g/L emerges as a frequently referenced value [14, 17]. Thus, Wilms-MOPS medium [50] was modified with 15 g/L NaCl for an initial shake flask cultivation. MOPS buffer (pK_a_ 7.2) offers a high buffering capacity around pH 7.5, but does not increase the medium osmolality as much as e.g. phosphate buffer when added to the medium at the same buffering capacity [53]. A conceptual overview referencing this step is provided in Fig. S4. The online-monitored oxygen transfer rate (OTR, violet) and the respiratory quotient (RQ, black) during the cultivation of *V.* *natriegens* Vmax are shown in Fig. 1A. After a short acceleration phase during the first 1.75 h of cultivation, the culture starts to grow exponentially as indicated by the OTR. After 2.75 h of cultivation time, the OTR forms a plateau at approximately 70 mmol/L/h, which indicates an oxygen limitation [37, 38]. From 3.5 h onwards, the OTR decreases until it reaches 0 mmol/L/h after 4.25 h. Initially, the RQ takes a value of about 1, which indicates aerobic growth [54]. During the oxygen limitation phase, the RQ drops below 1, indicating the formation of an oxidized component [55].

The optical density (OD_600_) and pH measurements in Fig. 1B support and extend the indications from the online data. The OD_600_ follows the exponential course of the OTR up to OD_600_ = 7.6 and then increases linearly to its maximum value of OD_600_ = 13 after 3.5 h, while the cultivation experiences an oxygen limitation (see Fig. 1A). As the OTR decreases, the OD_600_ decreases slightly and then rapidly to 9.8 at the end of cultivation. The pH curve progresses inversely to the OD_600_ curve. Starting from a pH of 7.2 of the inoculated medium, the pH decreases exponentially to 6.6 after 3.25 h and reaches 5.3 after 4 h. The pH then remains stable at 5.3 until the end of the cultivation. The pH curve generally corresponds to the glucose consumption and acetate production shown in Fig. 1C. The production of acetate was observed almost immediately. With increasing biomass formation, the acetate-producing capacity increases; thus, the acetate concentration increases to reach a final value of 1.6 g/L at the end of cultivation. After 3 h, its effect on pH becomes increasingly visible. Simultaneously, glucose is consumed, and the concentration decreases rapidly from 20.7 g/L to 14 g/L after 3 h. At the end of the cultivation, 3.5 g/L glucose and 1.6 g/L acetate remain unused in the culture broth. To avoid this problem, in the next step, the concentration of the MOPS buffer was increased to stabilize the pH value in a range in which *V. natriegens* can take up the acetate again.

### Resolving the pH inhibition in microtiter plate scale

**Fig. 2:**
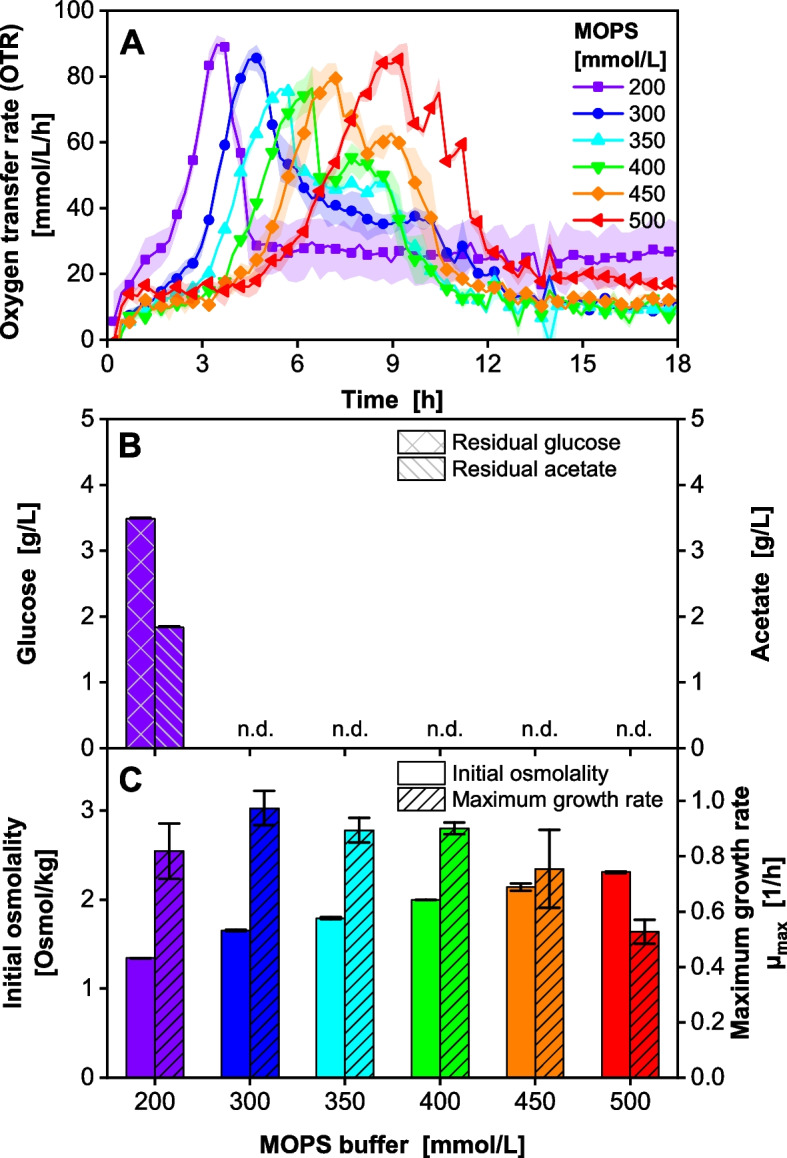
Influence of the MOPS buffer concentration on the medium osmolality and the maximum growth rate *V. natriegens* Vmax pET19b::LevS1417 in modified Wilms-MOPS medium (20 g/L glucose, 15 g/L NaCl, varied concentration of MOPS buffer set to pH 7.5). Initial OD_600_ 0.25, 37 °C, 50 µL filling volume in 96-DeepWell plate, 1000 rpm at 3 mm shaking diameter. **A** The oxygen transfer rate was monitored using a µTOM device. Mean of *n* = 2 replicates shown; shadows indicate minimum/maximum. For clarity, only every third data point is indicated by a symbol. **B** Residual glucose and acetate at the end of the cultivation, mean of *n* = 2 replicates, error bars indicate minimum/maximum and are barely visible due to the very low deviation. n.d. = not detected. **C** Initial osmolality of the medium at the cultivation start (left axis, empty bars) and maximum growth rate µ_max_ calculated from the OTR (right axis, shaded bars) as illustrated in Fig. S1 and S2. Mean of *n* = 2 replicates; error bars indicate minimum/maximum

For higher throughput, the following experiments were performed in 96-well MTPs, as illustrated in the conceptual overview provided in Fig. S4. The buffer was varied between 200 and 500 mM MOPS. The replicates were spread out across the parameter space as described in the Methods section and the previously determined biological variability of the maximum growth rate µ_max_ (Fig. S3) of 6.6% was applied to the data in all following experiments to estimate the respective errors. The OTR was monitored during the cultivation (Fig. 2A), and the residual glucose and acetate were determined at the end of the cultivation (Fig. 2B). The cultivation buffered with 200 mM MOPS initially progresses identically to the OTR during the shake flask experiment (Fig. 1A). After 2.75 h, the OTR does not indicate an oxygen limitation as seen in the shake flask experiment but increases further to up to 90 mmol/L/h. This is due to the different OTR_max_ values of the two culture systems [39, 51].

At the end of cultivation buffered with 200 mM MOPS, 3.5 g/L residual glucose and 1.8 g/L residual acetate were determined in the culture broth, which agrees well with the results of the shake flask experiment (Fig. 1C). As the concentration of MOPS buffer is stepwise increased, the OTR for the respective conditions increases later and later. At the same time, a higher MOPS buffer concentration stabilizes the pH in a non-inhibiting range. Consequently, *V. natriegens* is able to take the acetate up and metabolize it after glucose depletion. This phenomenon becomes visible as an OTR shoulder and is observed after the first OTR peak in all cultures with > 200 mM MOPS. As a result, for cultivations with > 200 mM MOPS, neither residual glucose nor acetate was detected in the endpoint samples.

Since the osmolality is defined as the total concentration of osmotically active particles per kg solvent (water), the medium osmolality in Fig. 2C (empty bars, left axis) increases linearly with the MOPS concentration from 1.3 Osmol/kg (200 mM MOPS) to 2.3 Osmol/kg (500 mM MOPS). The maximum growth rate in Fig. 2C (hatched bars, right axis) is 0.81 ± 0.05 1/h for the cultivation buffered with 200 mM MOPS. It increases to 0.97 ± 0.06 1/h for the cultivation buffered with 300 mM MOPS, as the pH inhibition due to unfavorable pH values is reduced. For higher MOPS concentrations, the maximum growth rate decreases steadily to 0.53 ± 0.03 1/h for the cultivation supplemented with 500 mM MOPS.

### Two-dimensional optimization of buffer and sodium concentration

**Fig. 3:**
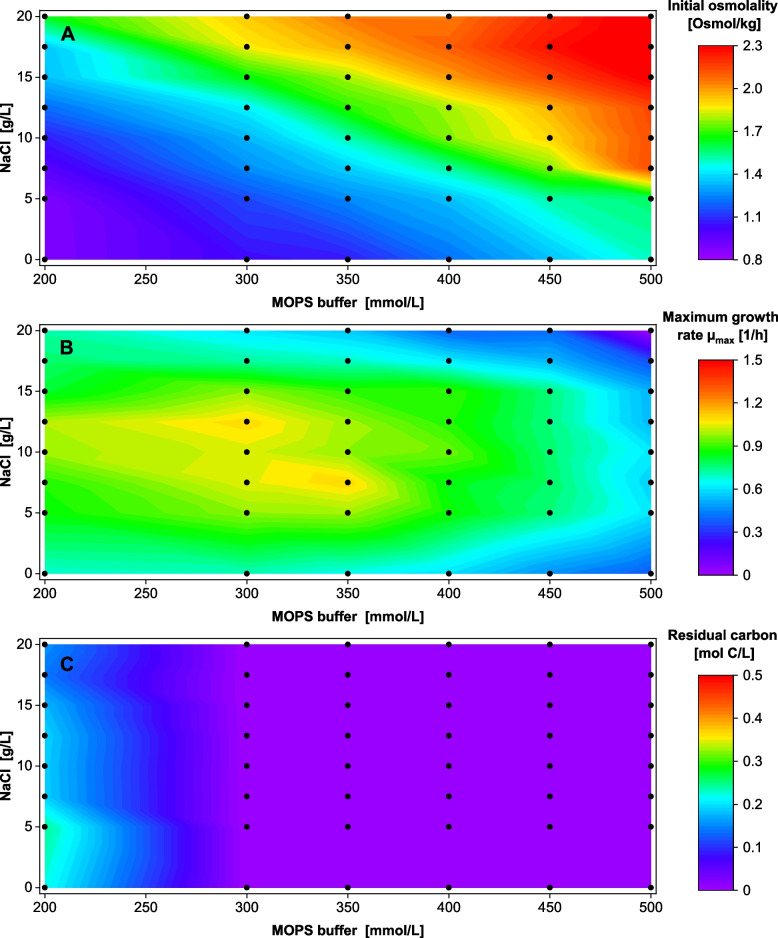
Medium osmolality, maximum growth rates, and residual carbon concentration for varied NaCl and MOPS concentrations *V. natriegens* Vmax pET19b::LevS1417 in modified Wilms-MOPS medium (20 g/L glucose, NaCl, and MOPS buffer concentration varied, MOPS buffer set to pH 7.5). Initial OD_600_ 0.25, 37 °C, 50 µL filling volume in 96-DeepWell plate, 1000 rpm at 3 mm shaking diameter. Measured conditions indicated by dots (mean of duplicates). **A** Osmolality of the medium at the cultivation start. **B** Maximum growth rate µ_max_ calculated from the OTR raw data in Fig. S5. **C** Residual carbon (glucose and acetate combined; c-molar) at the end of the cultivation

In the next step, the NaCl concentration was varied between 0 and 20 g/L in addition to the MOPS buffer variation from above (200–500 mM MOPS, see overview in Fig. S4). The complete data set of 96 OTR curves each is provided in Fig. S5. The online and offline results are summarized in Fig. 3 as heat maps of the resulting initial medium osmolality (A), maximum growth rate (B), and residual carbon (C). Black dots mark the experimental conditions that were tested. The lowest osmolality of 0.88 Osmol/kg was measured for the original Wilms-MOPS medium with 200 mM MOPS buffer and without any added NaCl (Fig. 3A). The highest osmolality measured was 2.3 Osmol/kg for the medium configuration of 500 mM MOPS and 20 g/L NaCl.

Figure 3B shows a growth rate optimum at 300–350 mM MOPS in combination with 7.5–12.5 g/L NaCl, where a growth rate of 1 ± 0.5 1/h was observed. While growth rates to the left of the optimum for lower buffer concentrations were only slightly lower, not all of the C source was consumed (Fig. 3C). However, glucose was completely consumed, and no residual acetate was detected at the end of cultivation for all medium configurations containing ≥ 300 mM MOPS. In the upper right corner of Fig. 3A and B, where the medium configurations with the highest osmolality are presented, a correlation between osmolality and growth becomes visible as a gradually decreasing growth rate. This is particularly evident for the maximum configuration of 500 mM MOPS with 20 g/L NaCl. Under these conditions, hardly any growth was detectable, an observation reflected in the time of glucose depletion (Fig. S6).

### Two-dimensional optimization at reduced osmolality and glucose and higher starting pH

To separate the effects of increased pH buffering and high osmolality, for the next experiments, all medium components, including glucose, were diluted to half their original concentration. Since for less available glucose, less detrimental effects on the pH were expected, the concentration window of the buffer was shifted to 180–400 mM MOPS, and testing was performed in smaller increments. The concentration window for NaCl was not changed (see illustration in Fig. S4).

**Fig. 4:**
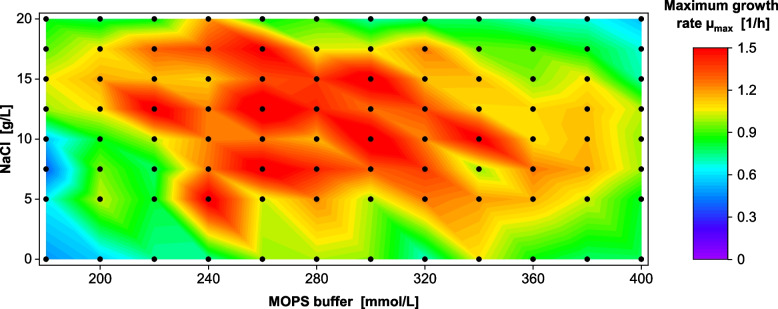
Maximum growth rates for cultivations in modified half-concentrated Wilms-MOPS medium *V. natriegens* Vmax pET19b::LevS1417 in modified half-concentrated Wilms-MOPS medium (10 g/L glucose, NaCl, and MOPS buffer concentration varied, MOPS buffer set to pH 7.5). Initial OD_600_ 0.25, 37 °C, 50 µL filling volume in 96-DeepWell plate, 1000 rpm at 3 mm shaking diameter. Measured conditions indicated by dots. Maximum growth rate µ_max_ calculated from the OTR raw data in Fig. S8

Figure 4 shows the resulting maximum growth rates (OTR raw data in Fig. S8, time of glucose depletion in Fig. S9, residual carbon in Fig. S10, start of the stationary phase in Fig. S11, and osmolality corresponds to the data set in Fig. S13). The lower osmolality experimental setup resulted in a much broader growth rate optimum of up to 1.6 ± 0.1 1/h (oval shape). A very low osmolality (bottom left corner) and too high osmolality (upper right corner) were associated with a decrease in growth rate.

**Fig. 5:**
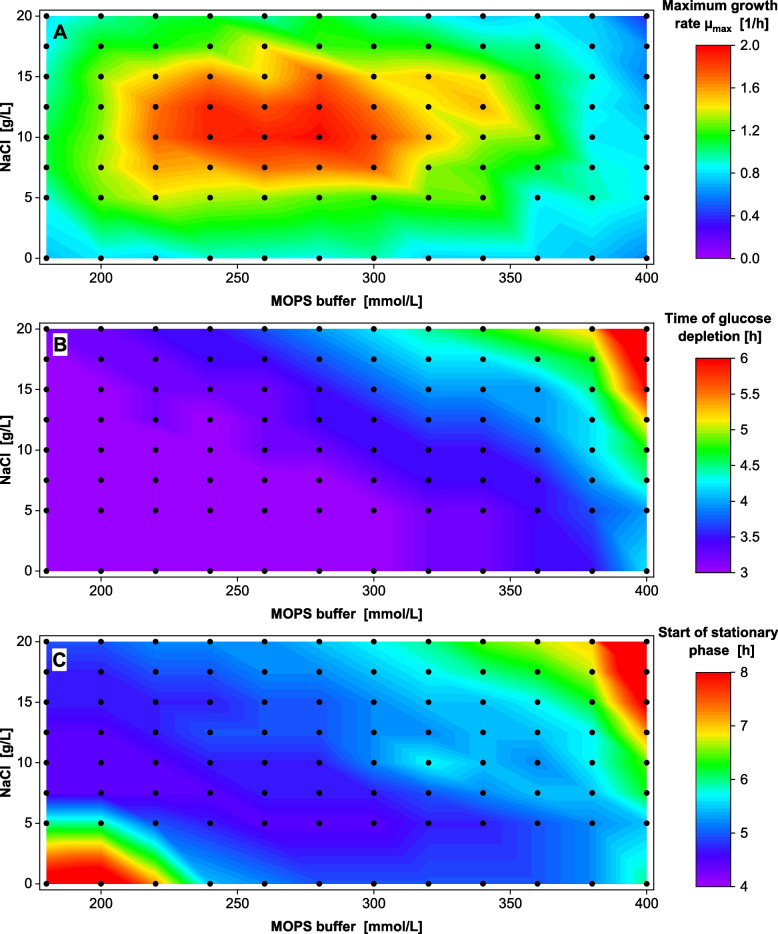
Varied NaCl and MOPS concentrations in modified half-concentrated Wilms-MOPS medium with higher initial pH = 8 *V. natriegens* Vmax pET19b::LevS1417 in modified half-concentrated Wilms-MOPS medium (10 g/L glucose, NaCl, and MOPS buffer concentration varied, MOPS buffer set to pH 8.0). Initial OD_600_ 0.25, 37 °C, 50 µL filling volume in 96-DeepWell plate, 1000 rpm at 3 mm shaking diameter. Measured conditions indicated by dots, color scale adjusted to data (not identical to Fig. 3 and 4). **A** Maximum growth rate µ_max_ calculated from the OTR raw data in Fig. S12. **B** Time of glucose depletion in hours, read out as the drop after the first OTR peak. **C** Start of the stationary phase in hours, determined from the OTR raw data (see illustration in Fig. S1)

In the final step, the initial pH of the medium was increased to 8.0 to fully exploit the MOPS buffer capacity. All remaining experimental conditions, including the half-concentrated medium composition, were kept constant. The growth rates were derived from the OTR raw data (Fig. S12) and are shown in Fig. 5A (note the adjusted color bar compared to the previous figures). This time, a defined growth rate optimum emerged between 7.5 and 15 g/L NaCl and 220—300 mM MOPS. The highest growth rate of 1.97 ± 0.13 1/h was measured on the 10 g/L NaCl and 280 mM MOPS medium configuration.

The medium osmolality ranged from 0.6 to 2.1 Osmol/kg (Fig. S13). The time of glucose depletion increased with increasing osmolality from 2.7 h on the 0 g/L NaCl/180 mM MOPS configuration to 7.6 h on the 20 g/L NaCl/400 mM MOPS medium configuration (Fig. 5B). At most medium configurations, the stationary phase (Fig. 5C) was reached after 4.5–5.5 h, approximately an hour earlier than on medium with a starting pH of 7.5 (Fig. S11). This means that not only the maximum growth rate on glucose increased (Fig. 5A), but the maximum growth rate was reached early within the first 1.5 h of the cultivation (Fig. S14) and remained high, which consequently reduced the process time by one hour. Only at high buffer and salt conditions (400 mM MOPS, > 15 g/L NaCl) as well as low buffer and salt conditions (< 200 mM MOPS and < 5 g/L NaCl), the maximum growth rate was only reached after > 2.5 h and a late onset of the stationary growth phase after 8–12 h was observed. No residual carbon was detected after the cultivations (Fig. S15).

## Discussion

In this study, we explored the cultivation conditions of *V. natriegens*, focusing on mineral medium compositions and osmolality to optimize its growth and uncover characteristics that may have implications for its utilization in bioprocessing.

### Influence of pH and buffer concentration on *V. natriegens*’ growth

Various mineral media, such as M9 [6, 27], MSM [28], and VN medium [17], have been proposed for the cultivation of *V. natriegens*. Depending on the medium, maximum growth rates of 1.36 1/h on MSM [28], 1.66 1/h on VN [17], and 1.7 1/h on M9 medium [27] were achieved at 37 °C. When *V. natriegens* was cultivated on Wilms-MOPS medium buffered with 200 mM MOPS, in this study, a maximum growth rate of 0.81 ± 0.05 1/h was observed (Fig. 1). Since glucose is consumed for biomass formation, the nitrogen source ammonia was taken up in a constant stoichiometric ratio, depending on the biomass composition. During the uptake, a proton was released because ammonia is provided as ammonium in Wilms-MOPS medium [50, 56]. This subsequently lowered the pH. In addition to that, due to glucose overflow metabolism, the organism produced large amounts of acetate [17, 32], which decreased the medium pH from 7.2 at the beginning of cultivation to a final pH of 5.3. The OD_600_ did not increase further once the pH dropped below 6.18 after 4 h cultivation time, leaving residual glucose and acetate in the culture broth at the end of cultivation.

The same phenomenon had previously been reported by Stella et al. [33] on VN medium with 20 -15 g/L glucose. Stella et al. [33] countered the effect by doubling the buffer concentration of VN medium from originally 100 mM to 200 mM MOPS. In this study, increasing the buffer capacity to 300—350 mM MOPS (20 g/L glucose, Fig. 3) resolved the pH inhibition and consequently allowed full carbon utilization, which led to an increased maximum growth rate of up to 1 ± 0.07 1/h. For medium variations with only 10 g/L glucose, 180 mM MOPS was sufficient, which agrees with Stella et al. [33]. These variations increased the maximum growth of *V. natriegens* rate up to 1.59 ± 0.10 1/h.

M9 and MSM medium, however, have an even lower relative buffer capacity than VN medium, as they only contain 96 mM or 13 mM phosphate buffer, respectively [6, 28]. Both media should only be employed with a pH regulating agent, as they are predestined for pH-inhibited cultivations of *V.* *natriegens* during pH-unregulated processes in, e.g., shake flasks and microtiter plates.

In addition to its acetate overflow metabolism, *V. natriegens* performs mixed acid fermentation and secretes mostly succinate, lactate, and formate under anaerobic and micro-aerobic conditions [17, 18]. For shaken cultures in flask and microtiter scale, this means that cultivation/shaking conditions must be carefully selected to provide sufficient oxygen and to avoid microaerobic conditions. In this study, a cultivation with 8 mL culture broth in 250 mL flasks (shaken at 350 rpm at a 50 mm shaking diameter) was oxygen-limited at approx. 70 mmol/L/h, which agrees with the theoretical value calculated after Meier et al. [51]. If oxygen is not provided sufficiently, mixed acid fermentation occurs, as observed by Hoffart et al. [17], and decreases the pH even faster than described above. By increasing the starting pH from the standard 6.5–7.5 [17, 27, 30] to pH 8.0, the necessary amount of buffer could be reduced, resulting in increased growth rates of up to 1.97 ± 0.13 1/h (Fig. 5).

### Influence of the time interval of the growth rate calculation

On Brain heart infusion (BHI), Eagon [1] calculated the generation time of *V. natriegens* over a 15 min interval during exponential growth as 9.8 min. Hoffart et al. [17] observed a generation time of 9.4 min over an identical 15 min interval under very specific conditions (low cell density, sterile-filtered BHI, raw material from Oxoid Ltd.), corresponding to a maximum growth rate of 4.43 1/h. However, when the maximum growth rate was determined over an interval of 1 h, only a value of 2.70 1/h was derived [17]. Both studies determined these growth rates in shake flasks. Since the cultivation conditions are constantly changing during unregulated batch processes with regard to nutrient availability, osmolality, pH, etc., the growth rate changes as well [57, 58]. The phenomenon is exemplarily illustrated in Fig. S1. Thus, the period of cultivation time over which the maximum growth rate is calculated has a decisive influence on the result (compare Fig. S2). The calculation over identical 1 h time intervals is therefore necessary to compare the maximum growth rate of 1.97 ± 0.13 1/h on mineral medium observed in this study (Fig. 5) to the 2.70 1/h on complex medium reported by Hoffart et al. [17]. For calculations of the growth rate for periods of ≥ 1 h (≥ 5 measurement values), only little deviation of the values derived was found. When recalculating for periods of less than 1 h of cultivation time, however (< 4 measurement values), even higher maximum growth rates of 2.05 1/h to 3.04 1/h can be derived from the experiment in Fig. 5 (see Fig. S2).

### Effects of sodium and medium osmolality on growth

As a marine organism, *V.* *natriegens* is presumed to require high NaCl concentrations for growth, attributed to sodium-dependent respiratory/membrane pumps [29]. The literature suggests varying NaCl concentrations from 15 g/L [17] and 17 g/L [14] up to 25 g/L [9] for cultivations. Generally, growth rates of 1.2–1.7 1/h [17, 27] are reported. The results in this work indicate an even lower concentration optimum between 7.5 and 15 g/L NaCl and suggest that the requirement may partially be due to increased osmolality rather than sodium dependency. In a genome-scale model, Coppens et al. [29] calculated a growth rate of 1.66 1/h at a salinity of 300 mM, which corresponds to 17.5 g/L NaCl. The results in Fig. 5 (200 – 220 mM MOPS and 17.5 g/L NaCl) match this value well.

This study explored a wide range of medium compositions and, consequently, osmolalities. It is well known that halophilic organisms require a certain osmolality/osmotic pressure to maintain their osmotic equilibrium [59–61]. Depending on the initial pH of the medium, an osmolality optimum for *V. natriegens* was identified between 1.0 and 1.4 Osmol/kg (pH_start_ 8, Fig. 5) or 1.1 and 1.5 Osmol/kg (pH_start_ 7.5, Fig. 4). Even at high osmolalities > 2 Osmol/kg, where e.g. *E. coli* experiences a severe osmotic shock [62], *V.* *natriegens* exhibited continued growth. This correlation between the growth rate and the respective salt concentration can be modeled as substrate inhibition kinetics [59] or as modified Arrhenius equations that take the water activity (derived from the osmolality) into account [52, 59, 63, 64]. Generally, the results revealed a clear correlation between osmolality and maximum growth rate in agreement with Biener et al. [30], who theorized that sodium and osmolality are separate influential variables for *V.* *natriegens*’ growth.

### Growth rate and space–time-yield

When designing a mineral medium, it is crucial to consider factors beyond maximum growth rates, such as carbon uptake kinetics. The results of this study indicate that depending on buffering conditions, slightly lower growth rates may coincide with faster reuptake of overflow metabolites and, thus, total carbon uptake (see Fig. 5C). Therefore, choosing an optimal medium composition is application-dependent—if high maximum growth rates are favored, then the half-concentrated medium supplemented with 10 g/L NaCl and 280 mM MOPS buffer (containing 10 g/L glucose, see Fig. 5A) should be chosen. It enables fast biomass generation for e.g. initial protein expression experiments or insight into the proteomics and metabolomics of fast growth. On the other hand, fast carbon uptake is a priority for time-efficient production processes where an increased space–time-yield is the aim. In this case, the medium configuration with 10 g/L NaCl and 450 mM MOPS buffer (containing 20 g/L glucose, see Fig. S7) is recommended, as this configuration led to the earliest onset of the stationary growth phase and consequently the highest space–time-yield. The understanding of growth dynamics, depending on the medium configuration, is essential for harnessing the full potential of *V.* *natriegens* in biotechnological applications.

## Conclusions

This study on the influence of the medium composition on *Vibrio natriegens*’ growth offers practical guidelines for laboratory cultivation in settings without pH and dissolved oxygen tension (DOT) regulation, such as microtiter plates or shake flasks. The findings illustrate the critical role of the pH, as *V. natriegens* secretes organic acids that lead to a pH inhibition when the medium is not sufficiently buffered. The literature suggests optimum sodium supplementation for *V. natriegens* ranging from 15 to 25 g/L NaCl. However, this study reveals a global optimum within a lower range of 7.5 to 15 g/L NaCl, a finding particularly relevant for industrial applications, where corrosion may be an issue. An osmolality optimum for *V. natriegens'* growth was observed between approximately 1 and 1.6 Osmol/kg. This prompts the assumption that the (local) NaCl optima reported in the literature may be attributed to a mixed consideration of sodium and osmolality influences. The optimization steps undertaken in this study resulted in a growth rate of 1.97 ± 0.13 1/h at 37 °C, to our knowledge the highest published growth rate for *V. natriegens* on a mineral medium. For future research, a similar approach is recommended to efficiently investigate the influence of different ions (e.g., K^+^ and Ca^2+^) and further carbon sources on *V.* *natriegens*.

## Supplementary Information

Supplementary Material 1. 

## Data Availability

The datasets supporting the conclusions of this article are included within the article and its additional file.

## Abbreviations

µTOM: Micro(µ)-scale transfer rate online measurement device
BHI: Brain heart infusion
CTR: Carbon dioxide transfer rate
DOT: Dissolved oxygen tension
MOPS: 2 M 3-(*N*-Morpholino)-propane sulfonic acid
MSM: Mineral salt medium
MTP: Microtiter plate
OD_600_: Optical density at 600 nm wavelength
OTR: Oxygen transfer rate
RAMOS: Respiration activity monitoring system
RQ: Respiratory quotient
