# Supplementary material for: Unraveling the impact of pH, sodium concentration, and medium osmolality on Vibrio natriegens in batch processes
Source: BMC Biotechnol. 2024 Sep 23;24:63. doi: 10.1186/s12896-024-00897-8 (PMC11421182; doi:10.1186/s12896-024-00897-8)
Supplement: Supplementary file 1 — Supplementary Material 1. [file 12896_2024_897_MOESM1_ESM.pdf]

Supplementary data

Unraveling the impact of pH, sodium concentration, and medium osmolality on *Vibrio natriegens* in batch processes

Eva Christine Forsten[1], Steffen Gerdes[1], René Petri[1], Jochen Büchs[1], Jørgen Barsett Magnus[1]

[1] AVT- Biochemical Engineering, RWTH Aachen University, Aachen, Germany

Correspondence: Prof. Dr.-Ing. Jørgen Barsett Magnus (Jorgen.Magnus@avt.rwth-aachen.de)

AVT - Biochemical Engineering, RWTH Aachen University, Forckenbeckstraße 51, 52074 Aachen, Germany.

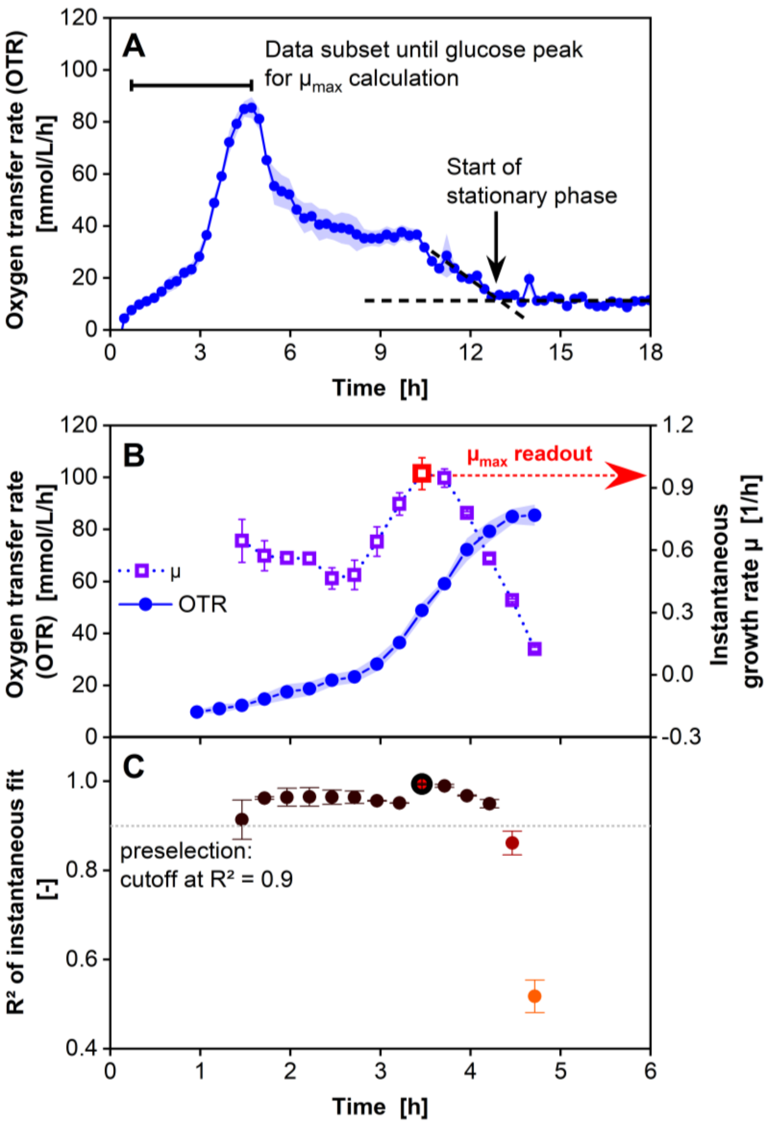

Figure S1: Illustration of the maximum growth rate calculation and stationary phase determination from the OTR. *V. natriegens* Vmax pET19b::LevS1417 in modified Wilms-MOPS medium (20 g/L glucose, 15 g/L NaCl, 300 mM MOPS buffer set to pH 7.5). Initial OD_{600} 0.25, 37°C, 50 µL filling volume in 96-DeepWell plate, 1000 rpm at 3 mm shaking diameter. Mean of n = 2 replicates shown, shadows/error bars indicate min/max. **(A)** Oxygen transfer rate over time for the full experiment duration. First measurement point (red) after OTR peak on glucose marks the time of glucose depletion. **(B)** Data subset until the glucose peak (as indicated in A). Left axis: OTR (blue dots). Right axis: Instantaneous growth rate calculated as a sliding fit over 5 values (violet squares). Highest obtained value (red symbol) was read out as the maximum growth rate μ_{max} of this experiment (see Fig. S2). **(C)** R^2 values obtained for the fit shown in **(B)**, only fits with an $R^2 > 0.9$ (marked black) were considered for the μ_{max} readout. More data from the same experiment is shown in Fig. 2, 3, S4, S5 and S6.

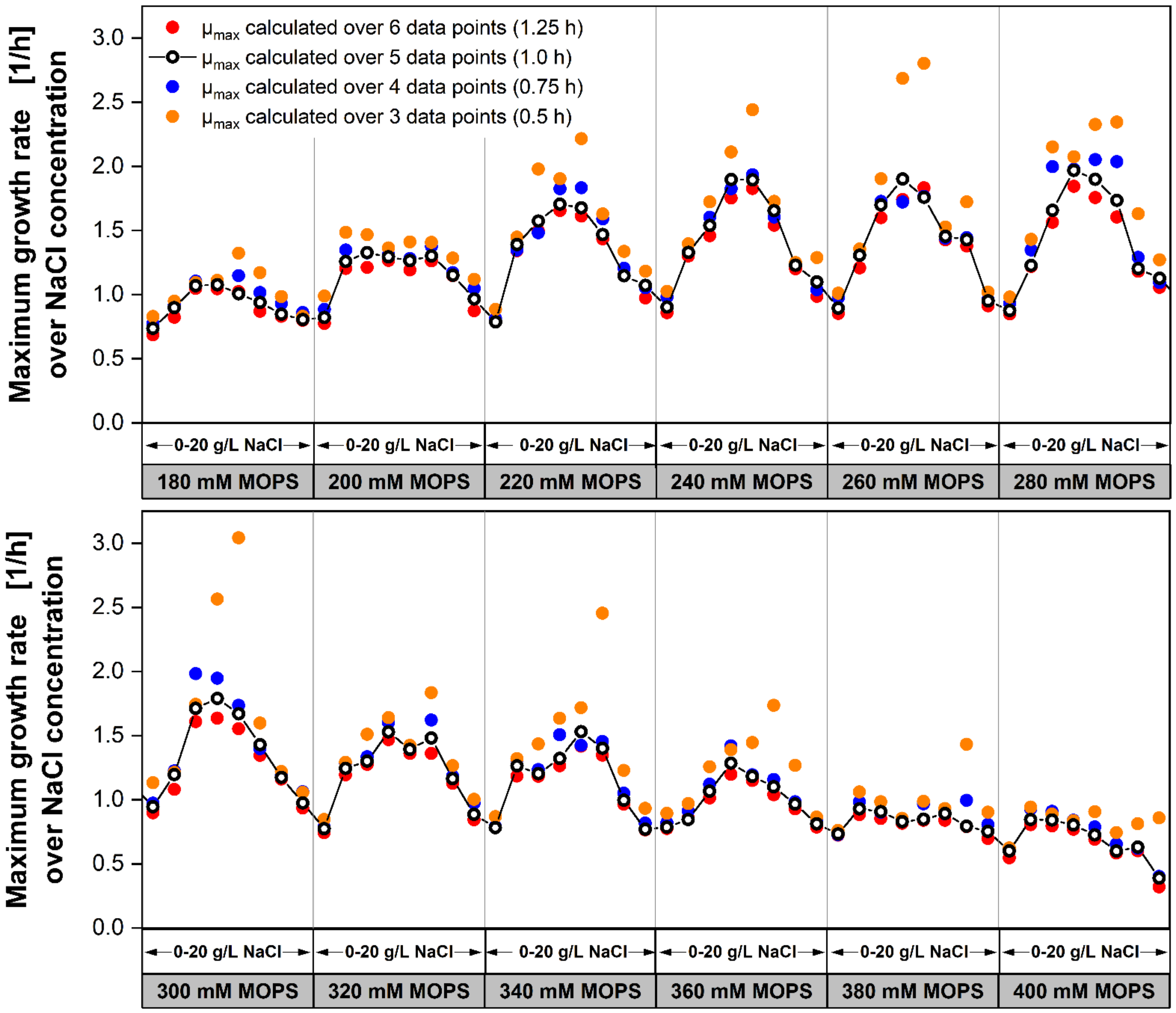

Figure S2: Absolute value of the maximum growth rate depending on the period of cultivation time included in the fit. *V. natriegens* Vmax pET19b::LevS1417 in modified half-concentrated Wilms-MOPS medium (10 g/L glucose, MOPS buffer set to pH 8.0). Y-axis: μ_{max} calculated as a sliding fit using three durations (0.75, 1.0, 1.25 and 1.5 h) for each microtiter plate experiment. X-axis: Grey segments group experiments with the same MOPS buffer concentration. Within the groups, experiments are ordered from 0 g/L (left) to 20 g/L (right) NaCl supplementation. More data from the same experiment is shown in Fig. 5, Fig. S11 (raw OTR data), S12, S13 and S14.

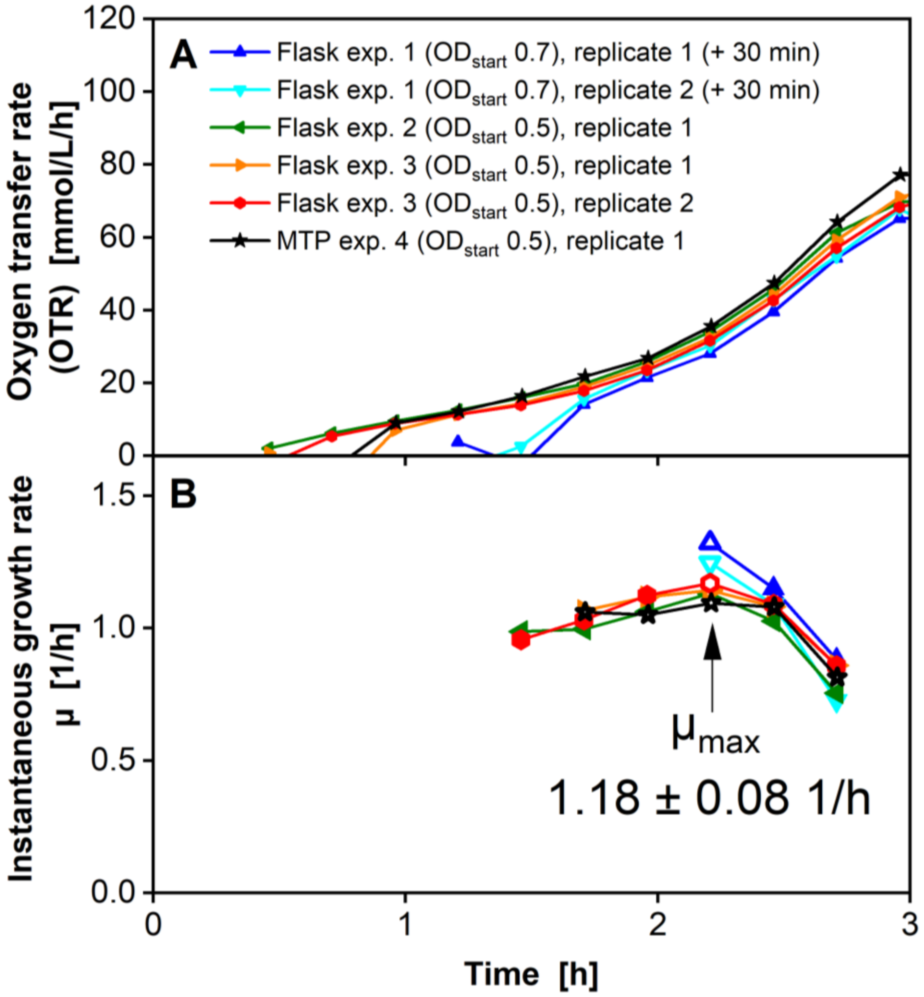

Figure S3: Investigating the biological variability of the *Vibrio natriegens* strain used in this work. *V. natriegens* Vmax pET19b::LevS1417 in modified Wilms-MOPS medium (7.5 g/L NaCl, 20 g/L glucose, MOPS buffer set to pH 7.5). Flask experiments: 37°C, 8 mL filling volume in 250 mL flask, 350 rpm at 50 mm shaking diameter. MTP experiment: 37°C, 50 µL filling volume in 96-DeepWell plate, 1000 rpm at 3 mm shaking diameter. **(A)** Oxygen transfer rate monitored using a RAMOS device (flask experiments) or a µTOM device (MTP experiments). Data stems from four different experiments with independent precultures (seed trains) conducted over a period of six months. Replicates denote parallel cultivations inoculated from the same preculture. Due to the higher inoculum density, data from the experiment inoculated with OD_{start} 0.7 is shifted backwards by 30 min on the x-axis. **(B)** Instantaneous growth rate µ calculated as a sliding fit using five OTR data points over time for each µ value. The maximum growth rate μ_{max} is then obtained as the highest overall µ value (empty symbol) (see method in Fig. S1). A slight influence of the inoculum becomes visible, as cultures inoculated with a lower density reach the maximum growth phase 30 min later. Overall, a value of 1.18 ± 0.08 1/h is derived, which corresponds to a relative deviation of 6.6 %.

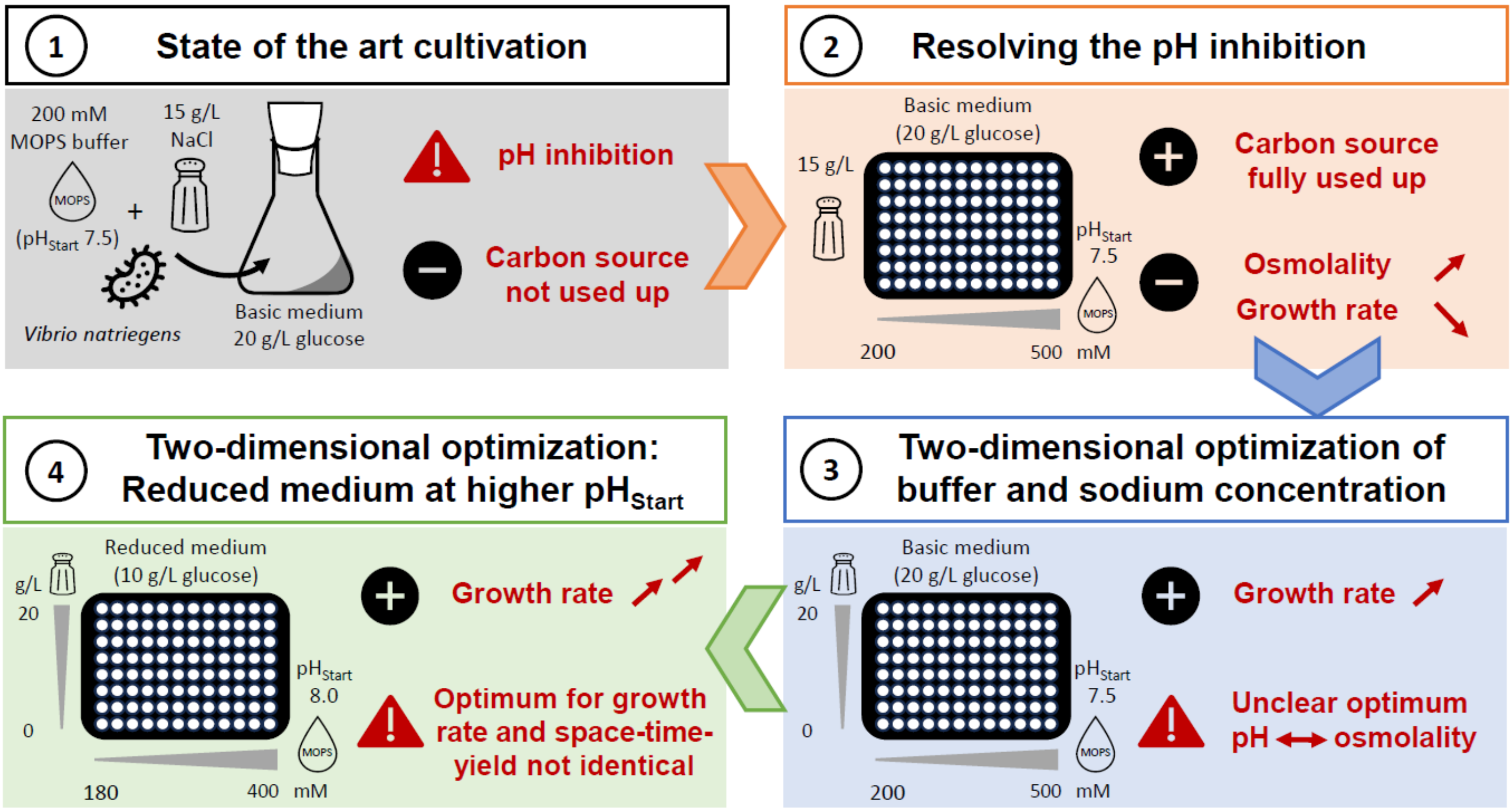

Figure S4: Concept of the research process.
Headings relate to the respective subheadings in the Results section. For context to Fig. 1 see section 1 (grey), for Fig. 2 see section 2 (orange), for Fig. 3 see section 3 (blue), and for Figs. 4 and 5 see section 4 (green).

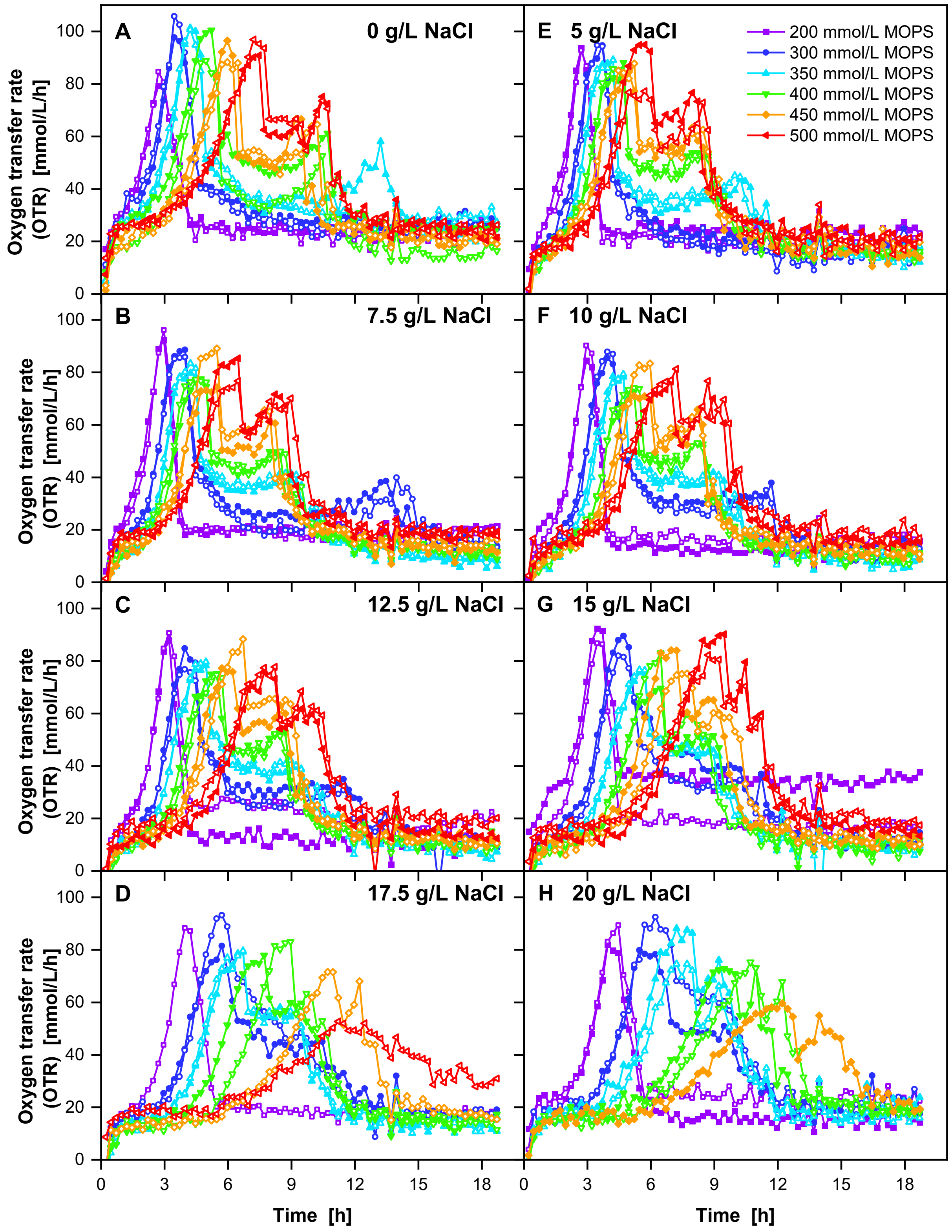

Figure S5: Complete OTR raw data corresponding to the data shown in Fig. 3. *V. natriegens* Vmax pET19b::LevS1417 in modified Wilms-MOPS medium (20 g/L glucose, NaCl and MOPS buffer concentration varied, MOPS buffer set to pH 7.5). Initial OD_{600} 0.25, 37°C, 50 µL filling volume in 96-DeepWell plate, 1000 rpm at 3 mm shaking diameter. n = 2 replicates. More data from the same experiment is shown in Fig. 2, 3, S1, S6 and S7. **(A)** 0 g/L NaCl, **(B)** 7.5 g/ L NaCl, **(C)** 12.5 g/L NaCl, **(D)** 17.5 g/L NaCl, **(E)** 5 g/L NaCl, **(F)** 10 g/L NaCl, **(G)** 15 g/L NaCl, **(H)** 20 g/L NaCl.

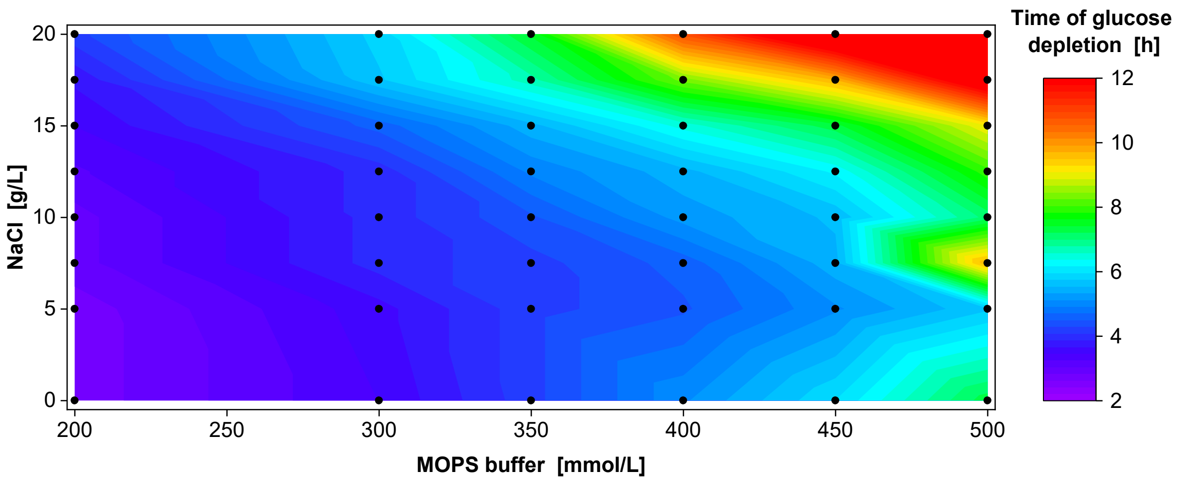

Figure S6: Time of glucose depletion derived from the drop after first OTR peak (as illustrated in Fig. S1A). *V. natriegens* Vmax pET19b::LevS1417 in modified Wilms-MOPS medium (20 g/L glucose, NaCl and MOPS buffer concentration varied, MOPS buffer set to pH 7.5). Initial OD_{600} 0.25, 37°C, 50 µL filling volume in 96-DeepWell plate, 1000 rpm at 3 mm shaking diameter. Measured conditions indicated by dots, mean of n = 2 replicates shown. More data from the same experiment is shown in Fig. 2, 3, S1, S5 (raw OTR data), and S7.

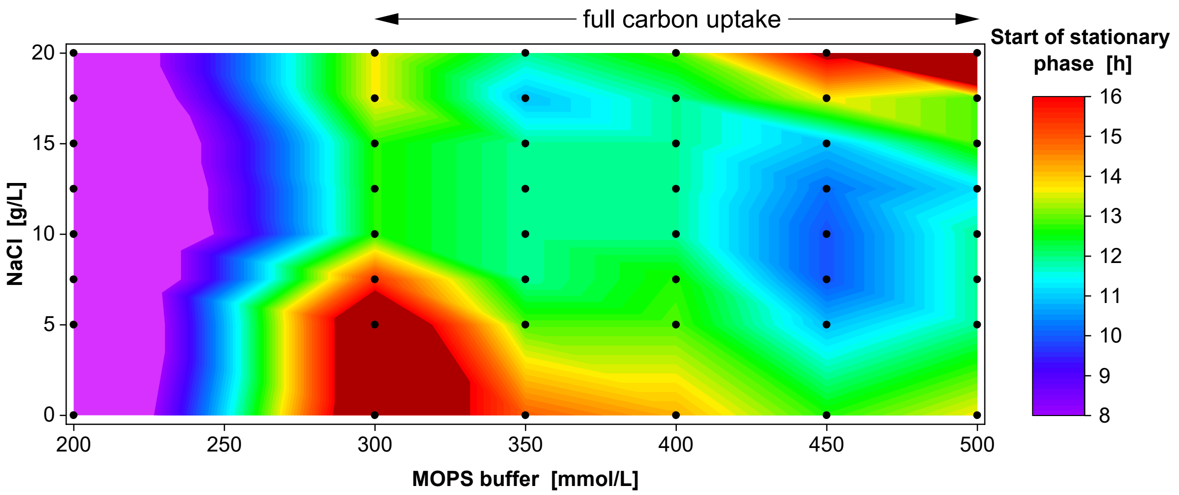

Figure S7: Start of the stationary phase, determined from the OTR (illustrated in Fig. S1A). *V. natriegens* Vmax pET19b::LevS1417 in modified Wilms-MOPS medium (20 g/L glucose, NaCl and MOPS buffer concentration varied, MOPS buffer set to pH 7.5). Initial OD_{600} 0.25, 37°C, 50 µL filling volume in 96-DeepWell plate, 1000 rpm at 3 mm shaking diameter. Measured conditions indicated by dots, mean of n = 2 replicates shown. More data from the same experiment is shown in Fig. 2, 3, S1, S5 (raw OTR data) and S6.

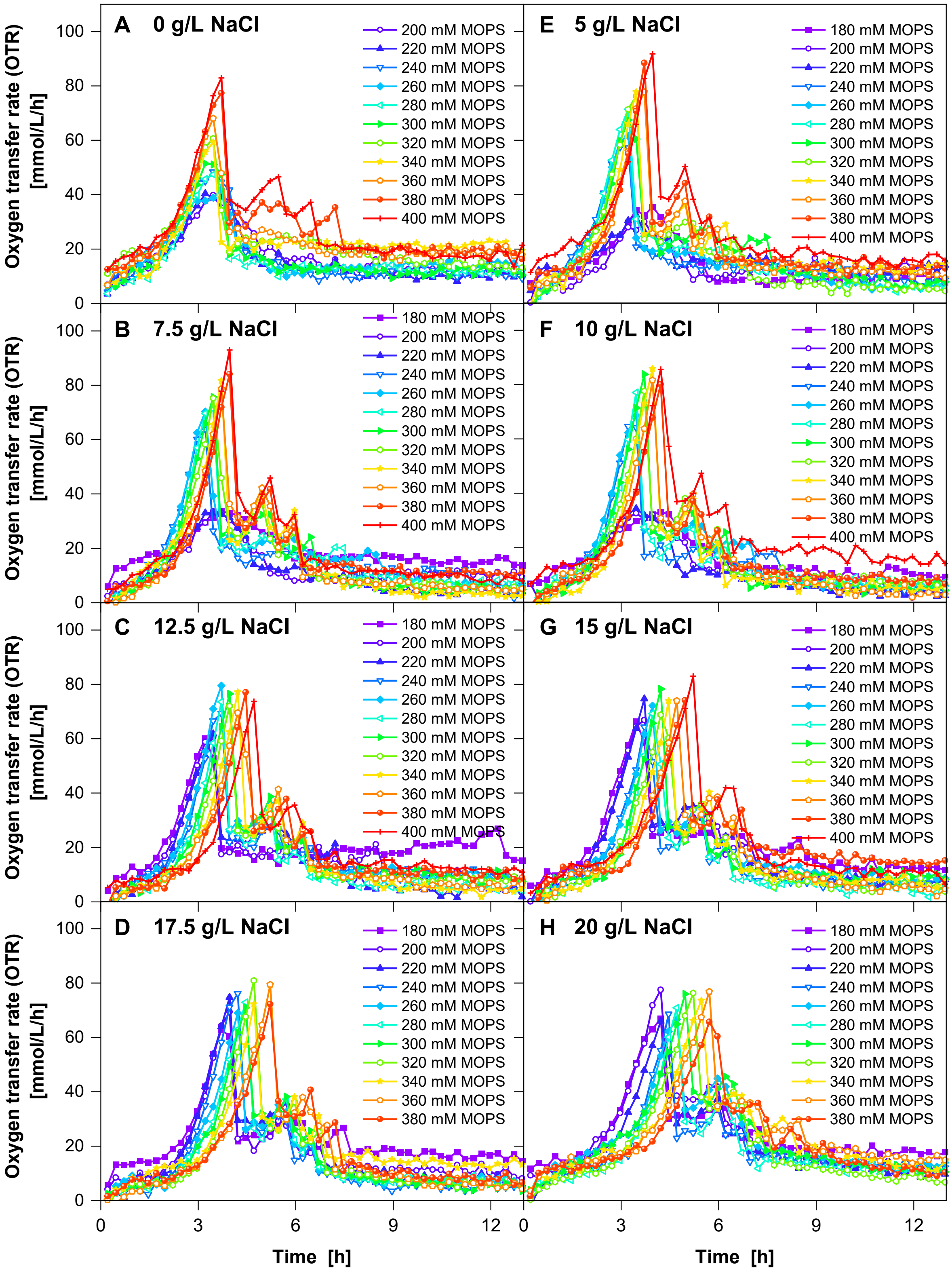

Figure S8: Full OTR raw data corresponding to the data shown in Fig. 4. *V. natriegens* Vmax pET19b::LevS1417 in modified half-concentrated Wilms-MOPS medium (10 g/L glucose, NaCl and MOPS buffer concentration varied, MOPS buffer set to pH 7.5). Initial OD_{600} 0.25, 37°C, 50 µL filling volume in 96-DeepWell plate, 1000 rpm at 3 mm shaking diameter. Oxygen transfer rate monitored using a µTOM device. More data from the same experiment is shown in Fig. 4, S9, S10 and S11. **(A)** 0 g/L NaCl, **(B)** 7.5 g/ L NaCl, **(C)** 12.5 g/L NaCl, **(D)** 17.5 g/L NaCl, **(E)** 5 g/L NaCl, **(F)** 10 g/L NaCl, **(G)** 15 g/L NaCl, **(H)** 20 g/L NaCl.

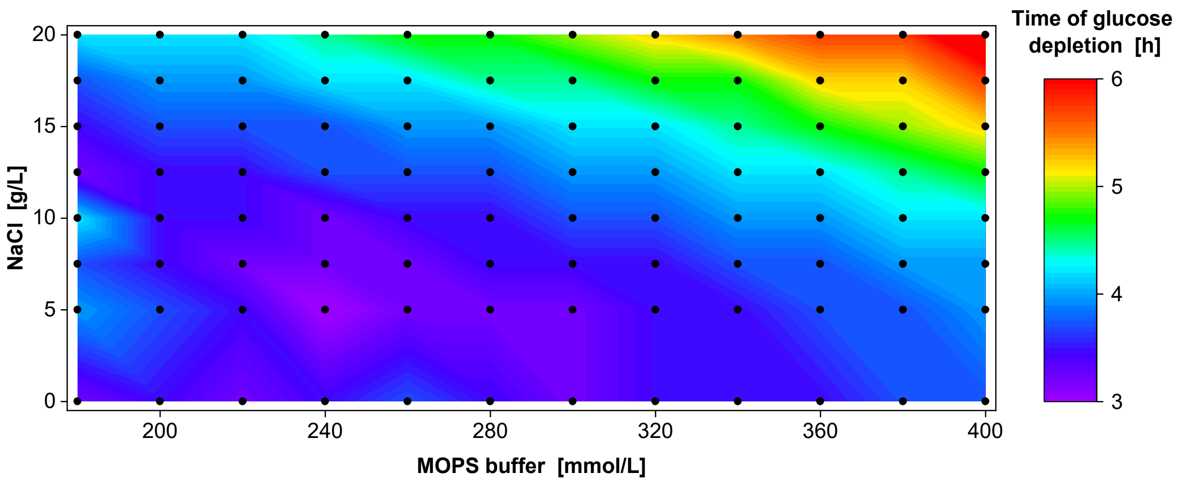

Figure S9: Time of glucose depletion derived from the drop after first OTR peak (as illustrated in Fig. S1A). *V. natriegens* Vmax pET19b::LevS1417 in modified half-concentrated Wilms-MOPS medium (10 g/L glucose, NaCl and MOPS buffer concentration varied, MOPS buffer set to pH 7.5). Initial OD_{600} 0.25, 37°C, 50 µL filling volume in 96-DeepWell plate, 1000 rpm at 3 mm shaking diameter. Measured conditions indicated by dots, mean of duplicates shown. More data from the same experiment is shown in Fig. 4, S8 (raw OTR data), S10 and S11.

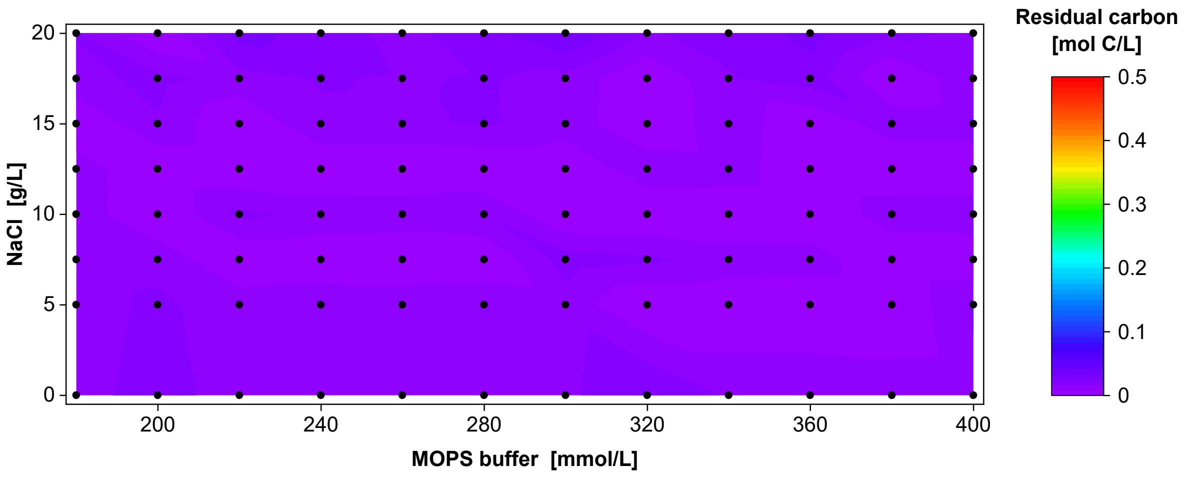

Figure S10: Residual carbon (glucose and acetate combined; c-molar) at the end of the cultivation on modified half-concentrated Wilms-MOPS medium. *V. natriegens* Vmax pET19b::LevS1417 in modified half-concentrated Wilms-MOPS medium (10 g/L glucose, NaCl and MOPS buffer concentration varied, MOPS buffer set to pH 7.5). Initial OD_{600} 0.25, 37°C, 50 µL filling volume in 96-DeepWell plate, 1000 rpm at 3 mm shaking diameter. Measured conditions indicated by dots, mean of duplicates shown. More data from the same experiment is shown in Fig. 4, S8 (raw OTR data), S9 and S10.

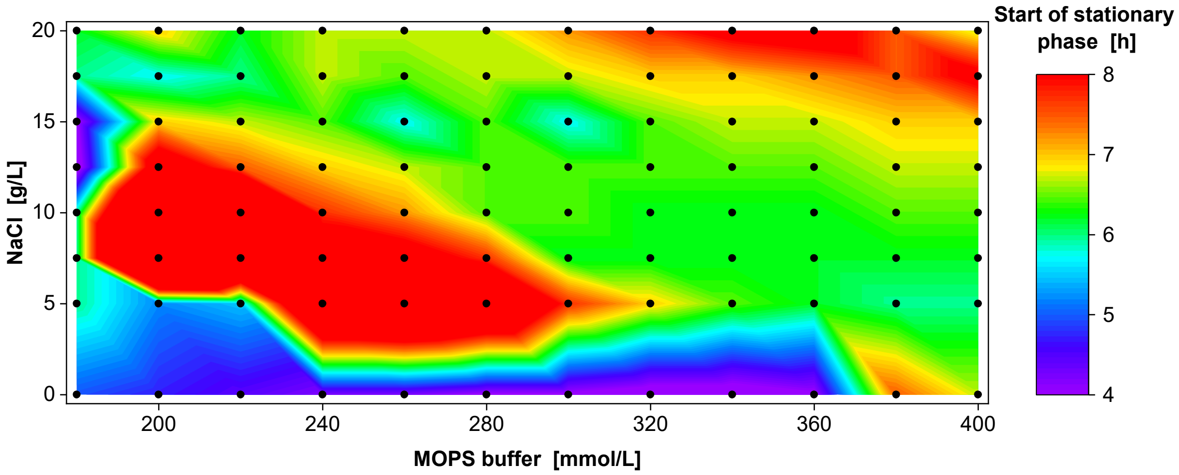

Figure S11: Start of the stationary phase, determined from the OTR (illustrated in Fig. S1A). *V. natriegens* Vmax pET19b::LevS1417 in modified half-concentrated Wilms-MOPS medium (10 g/L glucose, NaCl and MOPS buffer concentration varied, MOPS buffer set to pH 7.5). Initial OD_{600} 0.25, 37°C, 50 µL filling volume in 96-DeepWell plate, 1000 rpm at 3 mm shaking diameter. Measured conditions indicated by dots, mean of duplicates shown. More data from the same experiment is shown in Fig. 4, S8 (raw OTR data), S9 and S10.

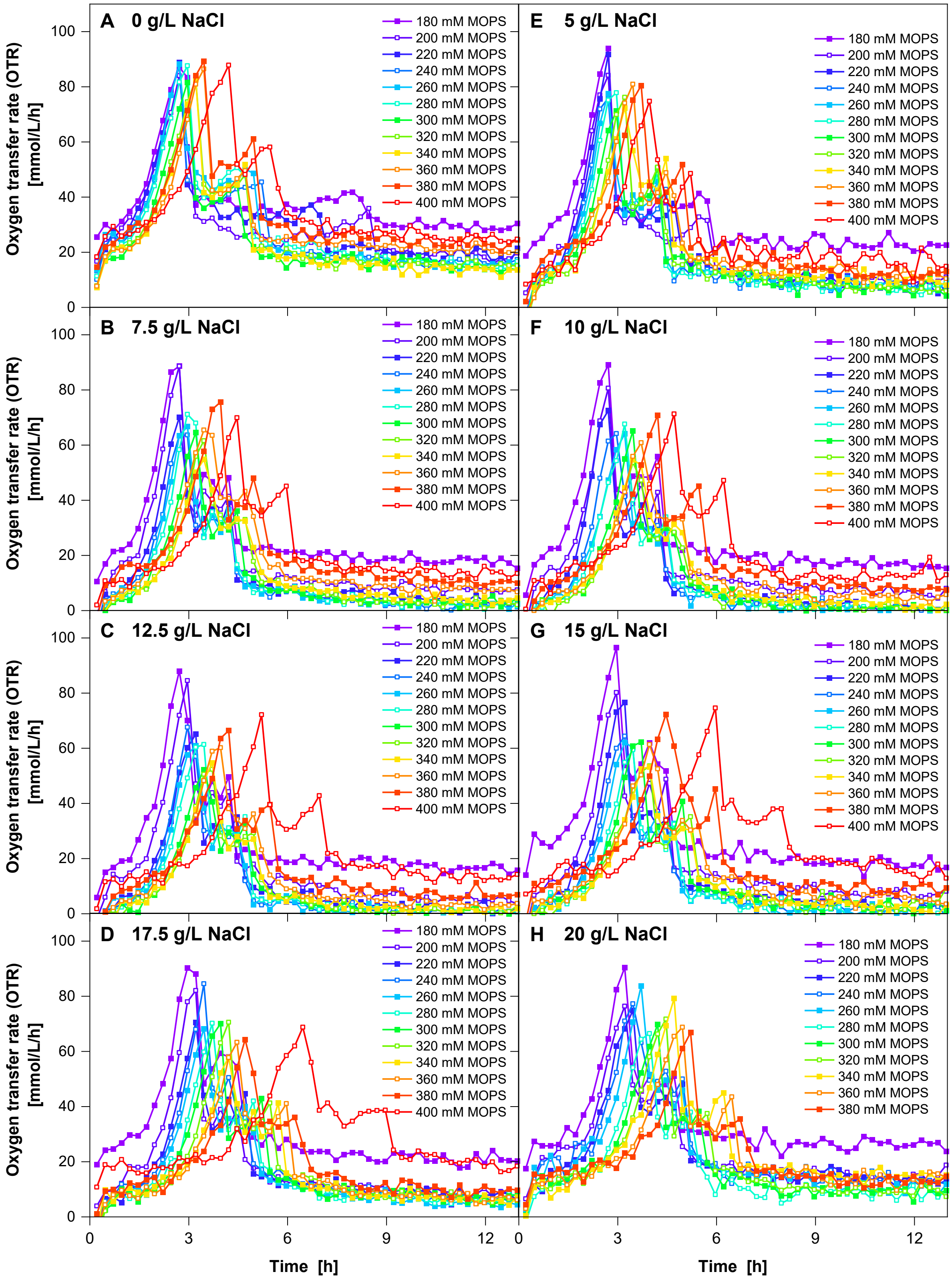

Figure S12: Full OTR raw data corresponding to the data shown in Fig. 5. *V. natriegens* Vmax pET19b::LevS1417 in modified half-concentrated Wilms-MOPS medium (10 g/L glucose, NaCl and MOPS buffer concentration varied, MOPS buffer set to pH 8.0). Initial OD_{600} 0.25, 37°C, 50 µL filling volume in 96-DeepWell plate, 1000 rpm at 3 mm shaking diameter. Oxygen transfer rate monitored using a µTOM device. More data from the same experiment is shown in Fig. 5, Fig. S2, S13, S14 and S15. **(A)** 0 g/L NaCl, **(B)** 7.5 g/ L NaCl, **(C)** 12.5 g/L NaCl, **(D)** 17.5 g/L NaCl, **(E)** 5 g/L NaCl, **(F)** 10 g/L NaCl, **(G)** 15 g/L NaCl, **(H)** 20 g/L NaCl.

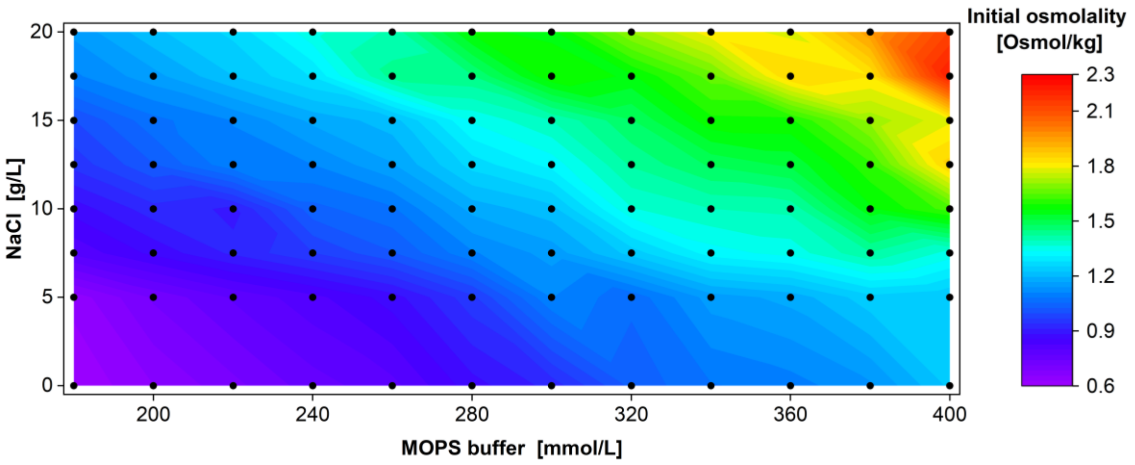

Figure S13: Initial osmolality of the modified half-concentrated Wilms-MOPS medium variations. *V. natriegens* Vmax pET19b::LevS1417 in modified half-concentrated Wilms-MOPS medium (10 g/L glucose, NaCl and MOPS buffer concentration varied, MOPS buffer set to pH 8.0). Initial OD_{600} 0.25, 37°C, 50 µL filling volume in 96-DeepWell plate, 1000 rpm at 3 mm shaking diameter. Measured conditions indicated by dots. More data from the same experiment is shown in Fig. 5, Fig. S2, S12 (raw OTR data), S14 and S15.

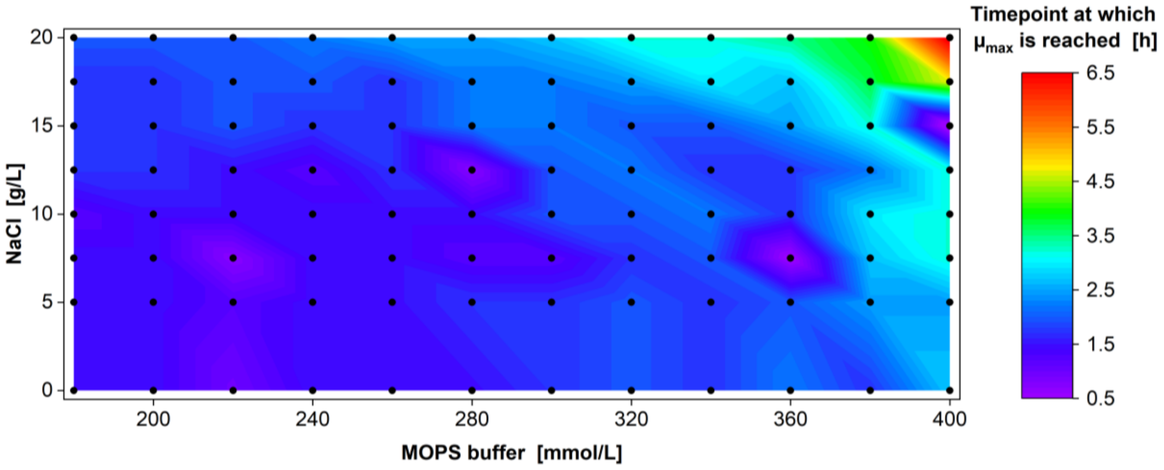

Figure S14: Time at which the cultures reach their respective maximum growth rate for cultivations on the modified half-concentrated Wilms-MOPS medium variations. *V. natriegens* Vmax pET19b::LevS1417 in modified half-concentrated Wilms-MOPS medium (10 g/L glucose, NaCl and MOPS buffer concentration varied, MOPS buffer set to pH 8.0). Initial OD_{600} 0.25, 37°C, 50 µL filling volume in 96-DeepWell plate, 1000 rpm at 3 mm shaking diameter. Measured conditions indicated by dots. More data from the same experiment is shown in Fig. 5, Fig. S2, S12 (raw OTR data), S13 and S15.

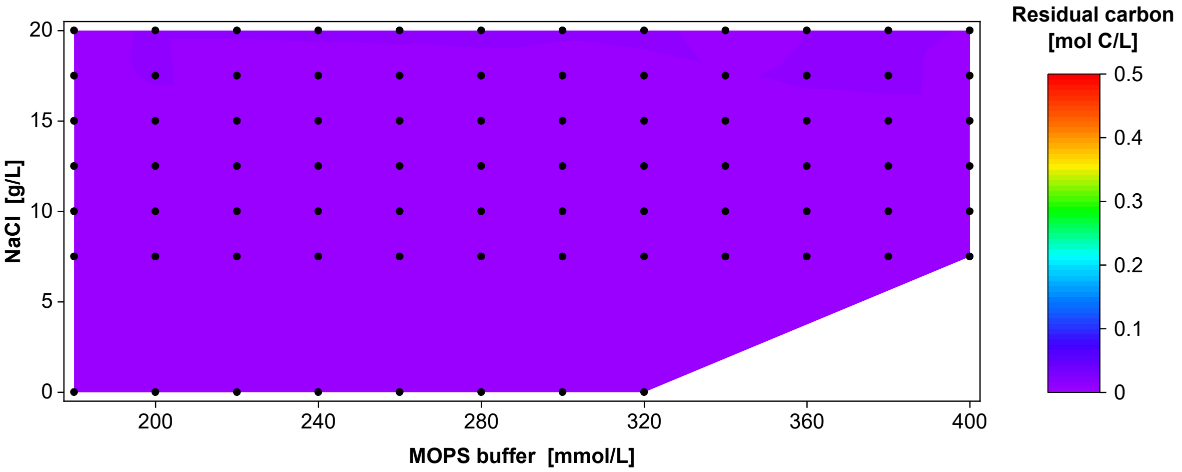

Figure S15: Residual carbon (glucose and acetate combined; c-molar) at the end of the cultivation on modified half-concentrated Wilms-MOPS medium with a higher initial pH of 8.0. *V. natriegens* Vmax pET19b::LevS1417 in modified half-concentrated Wilms-MOPS medium (10 g/L glucose, NaCl and MOPS buffer concentration varied, MOPS buffer set to pH 8.0). Initial OD_{600} 0.25, 37°C, 50 µL filling volume in 96-DeepWell plate, 1000 rpm at 3 mm shaking diameter. Measured conditions indicated by dots. More data from the same experiment is shown in Fig. 5, Fig. S2, S13 (raw OTR data), S13 and S14. Due to the very low sample volume, some conditions could not be HPLC-analyzed (white area).
